# Machine Learning-Driven Multi-Objective Optimization of Bead Geometry and Energy Efficiency in Laser–Arc Hybrid Additive Manufacturing

**DOI:** 10.3390/ma18245560

**Published:** 2025-12-11

**Authors:** Chunyang Xia, Kui Zeng, Jiawei Ning, Yaoyu Ding, Yonghui Liu

**Affiliations:** 1 College of Engineering, Ocean University of China, Qingdao 266100, China; chunyang_xia@sjtu.edu.cn; 2 School of Materials Science and Engineering, Shanghai Jiao Tong University, Shanghai 200240, China; 3 School of Mechanical and Electrical Engineering, China University of Petroleum, Qingdao 266580, China; 15871067632@163.com (K.Z.); 15632969132@163.com (J.N.); 4 School of Automation Engineering, University of Electronic Science and Technology of China, Chengdu 611731, China; yding@uestc.edu.cn; 5 Shandong Key Laboratory of Additive Manufacturing Technology & Equipment, Ocean University of China, Qingdao 266400, China

**Keywords:** additive manufacturing, laser–arc hybrid, bead geometry, energy efficiency, multi-objective optimization

## Abstract

Laser–arc hybrid additive manufacturing (LAHAM) combines the benefits of arc-based deposition and laser precision but involves complex, nonlinear process interactions that challenge the prediction and control of bead geometry and energy consumption. This study develops a machine learning (ML) framework to predict bead width, height, and Deposition volume per unit energy (DVUE) in LAHAM. Using experimental data, multiple regression models—including Support Vector Regression, Gaussian Process Regression, Neural Networks, and XGBoost—were trained and evaluated. Gaussian Process Regression (GPR) demonstrated superior performance in capturing nonlinear relationships and was further optimized using Bayesian Optimization and Particle Swarm Optimization. The optimized GPR models were integrated with the NSGA-II multi-objective optimization algorithm to simultaneously minimize geometric deviations and maximize DVUE. Results show that the proposed approach effectively identifies Pareto-optimal process parameters, achieving a balance between deposition accuracy and energy utilization rate, thereby providing a reliable and intelligent strategy for process optimization in hybrid additive manufacturing.

## 1. Introduction

In additive manufacturing processes, the introduction of auxiliary energy fields has gradually become an important emerging trend [1,2]. Laser–arc hybrid additive manufacturing (LAHAM) has emerged as a promising technique for fabricating large-scale metallic components, combining the high deposition efficiency of arc-based processes with the precision and energy concentration of laser heat sources [3,4]. By coupling the two energy inputs, LAHAM offers improved molten pool stability, enhanced material utilization, and greater flexibility in controlling thermal–mechanical behavior compared with conventional arc-based additive manufacturing [5,6]. Despite these advantages, the complex interaction between laser and arc heat sources leads to coupled and highly nonlinear process dynamics, which significantly complicates the prediction and control of bead geometry and energy efficiency [7,8,9]. Accurate prediction of deposition dimension—particularly bead width and height—together with quantitative evaluation of energy utilization, is essential for achieving stable manufacturing and optimizing process efficiency [10,11,12].

Traditional approaches to predicting bead morphology rely heavily on empirical models or physics-based simulations [13,14]. Empirical relations, although straightforward, often lack generalizability and fail to capture nonlinear interactions among process parameters [15,16]. High-fidelity numerical simulations provide deeper physical insights but are computationally expensive, making them difficult to integrate into real-time optimization or adaptive control frameworks [17,18]. With the rapid development of artificial intelligence, machine learning (ML) has become an effective means of learning process–property relationships directly from data, offering fast and accurate predictions for complex manufacturing processes [19,20,21]. Recent studies have demonstrated the potential of ML models in various additive manufacturing modalities [22,23], particularly in mapping process parameters to melt pool characteristics [24,25], thermal history [26,27], and mechanical properties [28]. However, research on ML-driven prediction in LAHAM remains limited, especially in jointly predicting bead dimensions and energy efficiency.

Recent developments in machine learning across materials and manufacturing science have demonstrated the increasing importance of data-driven prediction frameworks, robust optimization strategies, and model interpretability. For example, advanced ML pipelines have been used to identify critical biomarkers in complex biological systems through integrated feature learning [29], to enhance thermal error reduction in precision machine tools [30], and to develop interpretable combinatorial learning methods for evaluating geological or energy systems [31]. Similar ML-driven predictive models have also been applied in petroleum engineering for modeling nonlinear viscosity–temperature relationships [32], as well as in optimization of thermal–structural behavior in machine tool components [33]. These studies highlight the growing trend of using hybrid ML models to capture complex cross-domain physical interactions, combining prediction accuracy with interpretability and optimization. Building upon this broader progress, our study integrates regression-based surrogate modeling with multi-objective evolutionary optimization to address the coupled thermo-mechanical relationships in laser–arc hybrid additive manufacturing.

This study addresses these challenges by developing ML models to predict bead width, height, and DVUE (Deposition Volume per Unit Energy) in LAHAM. The proposed approach integrates experimentally collected process–response datasets with advanced regression algorithms to construct accurate predictive models that reflect the complex relationships among key process parameters. Furthermore, the predictions serve as objective functions in a multi-objective optimization framework, enabling the identification of parameter combinations that balance deposition accuracy and energy efficiency. By embedding ML-based prediction into the optimization workflow, this research establishes an efficient and scalable strategy for guiding process design in LAHAM.

Overall, this work contributes to the field in three aspects: (1) establishing a comprehensive experimental dataset that captures bead geometry and DVUE under varying laser–arc hybrid parameters conditions; (2) developing ML-based surrogate models capable of accurately predicting deposition outcomes in a highly coupled thermal process; and (3) enabling multi-objective optimization for simultaneous improvement of dimensional prediction accuracy and energy utilization. These contributions provide a foundation for intelligent control and sustainable operation of next-generation hybrid additive manufacturing systems.

## 2. Experimental System and Material

**Figure 1:**
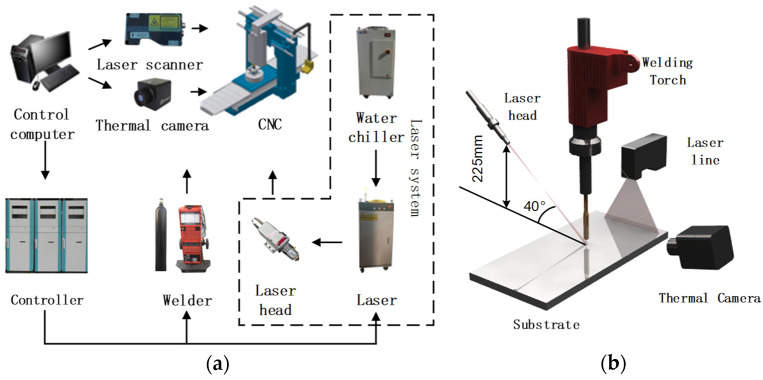
Overview of the laser–arc hybrid AM experimental system: (**a**) equipment setup. (**b**) hybrid energy source configuration.

In this study, a LAHAM system was developed to conduct process characterization and build-quality evaluations. The experimental setup consists of a fiber laser (IPG Photonics Corporation, Oxford, MA, USA) and an advanced pulsed Gas Metal Arc Welding (GMAW) unit (Fronius International GmbH, Wels, Austria). and a multi-axis motion control platform (as shown in Figure 1a). The configuration of the hybrid energy sources and sensors is presented in Figure 1b. The laser head is positioned at a 40° inclination relative to the substrate surface, with a standoff distance of 225 mm, while the welding torch is arranged nearly vertically to achieve effective hybridization of the laser and arc heat sources. A laser scanner (Micro-Epsilon Messtechnik GmbH & Co. KG, Ortenburg, Germany) was employed to capture the profile of the deposited beads. The entire system is equipped with a real-time monitoring module that enables precise regulation of process parameters and energy inputs, ensuring well-controlled experimental conditions and reliable results.

The substrate material used in this work is 2219-T6 aluminum alloy plates. This alloy offers high specific strength, excellent weldability, and superior fatigue resistance, making it a common structural material in aerospace applications and well-suited for fabricating lightweight, high-strength components via additive manufacturing. ER2319 aluminum alloy filler wire (1.2 mm diameter) was selected due to its close chemical compatibility with the 2219 series alloys. Under hybrid energy-field conditions, it provides strong metallurgical bonding and consistent weld–base metal matching in terms of microstructure and mechanical properties, establishing a solid foundation for subsequent build-quality assessment and machine-learning-based prediction tasks.

To reduce the influence of measurement uncertainties, each deposited bead was scanned three times using a calibrated laser scanning system (10 μm resolution, Micro-Epsilon Messtechnik GmbH & Co. KG, Ortenburg, Germany), and the averaged geometric parameters were used for model training. Energy inputs from the laser and GMAW units were recorded directly from the machine logs with manufacturer-specified uncertainties below ±2%. Because DVUE is computed as the ratio of deposition volume to total energy, the propagated uncertainty remained small and consistent across samples. Z-score normalization further reduced sensitivity to scale-related measurement noise during model fitting.

## 3. Machine Learning Modeling

### 3.1. Data Preprocessing

In this study, a total of 46 datasets were used for training and 20 datasets for validation, the detail of the dataset was presented in Appendix A and Appendix B. These datasets include five key process parameters—such as wire feed speed, laser power and travel speed—as well as three bead geometry indicators: bead width (W), bead height (H), and bead cross-sectional area (BCSA). In machine learning and data-driven analysis, data normalization is an essential preprocessing step. It eliminates differences in units and scales among features, prevents the model from being biased toward features with larger numerical magnitudes, and consequently improves both training efficiency and generalization performance.

The dataset consists of 66 experimental samples. A 70/30 split was adopted, resulting in 46 training samples and 20 testing samples. This ratio provides a balance between preserving enough samples to train the regression models while maintaining an independent test set for unbiased performance evaluation. Although cross-validation was considered, we employed 5-fold cross-validation during hyperparameter tuning to mitigate overfitting. The final evaluation was performed on the held-out test set to ensure independent assessment of predictive performance.

To ensure consistent feature scaling, this study employs Z-score normalization (standard score normalization) for both input features and output targets. This method transforms each variable into a distribution with zero mean and unit standard deviation, effectively accelerating model convergence and stabilizing the training process. For each sample $x_{i}$ in the input feature set *X*, the normalized value is computed as:

(1) $$x_{i}^{\prime }=\frac{x_{i}-\mu }{\sigma }$$

where μ and σ denote the mean and standard deviation of the training data, respectively. The normalization process is implemented using the StandardScaler class from the scikit-learn library, strictly following the principle that all data must be transformed using the statistical parameters computed from the training set. This ensures consistency between training and testing data preprocessing. The model predictions can be converted back to the original physical scale through an inverse transformation, expressed as:

(2) $$x_{i}=x_{i}^{\prime }\cdot \sigma +\mu$$

### 3.2. Model Training Strategy

**Figure 2:**
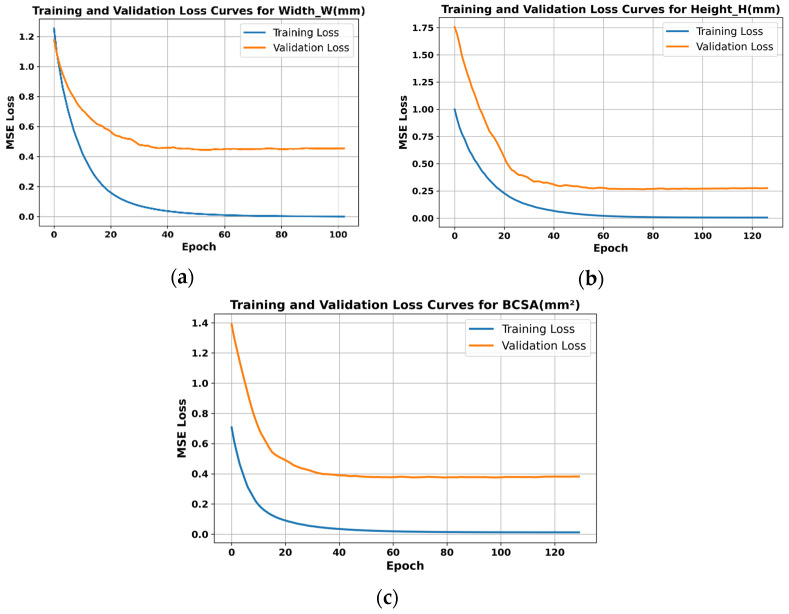
Training and validation loss curves of NN: (**a**) Width model. (**b**) Height model. (**c**) BCSA model.

To ensure reliable generalization and high prediction accuracy in modeling forming dimension and energy utilization for LAHAM, a systematic model training strategy was adopted. The dataset used in this study consists of 66 experimentally measured samples, of which 46 were used for training and 20 for testing. All five process parameters—wire-feed speed, travel speed, arc length correction, pulse correction, and laser power—were included as input features. Preliminary correlation analysis and variance checks confirmed that each parameter contributed non-redundant information and none exhibited near-zero variance; therefore, no dimensionality reduction or feature elimination was applied. Prior to model training, both the input features and output variables (bead width, bead height, and BCSA) were normalized using Z-score normalization. The mean and standard deviation were computed exclusively from the training set to avoid data leakage and ensure consistent preprocessing of the test set. Hyperparameters of multiple regression models were then optimized as follows:

1. Support Vector Regression (SVR): An RBF kernel–based SVR was employed, where the penalty parameter C(100–1000) and kernel coefficient γ(0.0001–0.01) were tuned via grid search to balance model complexity and fitting accuracy.
2. Gaussian Process Regression (GPR): A composite kernel (RBF × constant kernel + white-noise kernel) was used. Bayesian optimization was applied to identify optimal hyperparameters, including the length scale (1 × 10^−4^–1 × 10^4^) and noise level (0.1–1.0), with the Expected Improvement (EI) acquisition function accelerating global convergence.
3. Neural Network (NN): A multilayer perceptron (MLP) with two hidden layers (64 and 32 neurons) was developed using PyTorch 2.7.0. Training employed the Adam optimizer (learning rate = 0.001) and L2 regularization (weight decay = 1 × 10^−5^), while early stopping (patience = 200) was introduced to prevent overfitting. Training and validation loss curves are presented in Figure 2, demonstrating that validation loss plateaued earlier than training loss, confirming that early stopping effectively prevented overfitting.
4. XGBoost: The model parameters were set to 500 trees, a maximum depth of 5, and a learning rate of 0.01, with a regularization coefficient of 0.5 to control model complexity.
5. SVR (RBF kernel): C ∈ [100, 1000], γ ∈ [1 × 10^−4^, 1 × 10^−2^], ε ∈ [0.01, 0.2].
6. GPR: length-scale ∈ [1 × 10^−4^, 1 × 10^4^], noise level α ∈ [1 × 10^−6^, 1], kernels including RBF, Matern 1.5, and white-noise kernel combinations.

Model performance was evaluated using four metrics—$R^{2}$, MAE, MSE, and MAPE. To assess overfitting tendencies, the results on the training and validation sets were compared across all models. Based on the overall evaluation, the most suitable model was selected for subsequent prediction tasks.

### 3.3. Model Performance Evaluation

To quantitatively assess the performance of ML models in predicting forming dimension for laser-arc hybrid AM, this study employs multiple evaluation metrics to comprehensively measure model accuracy, stability, and generalization capability:

(a) Coefficient of Determination ($R^{2}$)

The coefficient of determination reflects the proportion of variance in the data explained by the model and is defined as:

(3) $$R^{2}=1-\frac{\sum_{i=1}^{n}{\left(y_{i}-\hat{y_{i}}\right)}^{2}}{\sum_{i=1}^{n}{\left(y_{i}-\bar{y}\right)}^{2}}$$

R^2^ ranges from 0 to 1, with values closer to 1 indicating better model fit. In this study, R^2^ serves as the primary reference for selecting the optimal model and evaluating improved models.

(b) Mean Absolute Error (MAE)

MAE measures the average absolute deviation between predicted and true values:

(4) $$\mathrm{MAE}=\frac{1}{n}\sum_{i=1}^{n}\left|y_{i}-\hat{y_{i}}\right|$$

MAE is robust to outliers and has the same unit as the output variable.

(c) Mean Absolute Percentage Error (MAPE)

MAPE expresses the relative error in percentage form, eliminating the influence of scale:

(5) $$\mathrm{MAPE}=\frac{1}{n}\sum_{i=1}^{n}\left|\frac{y_{i}-\hat{y_{i}}}{y_{i}}\right|\times 100\%$$

(d) Root Mean Square Error (RMSE)

RMSE reflects the dispersion of predictions and is more sensitive to large errors:

(6) $$\mathrm{RMSE}=\sqrt{\frac{1}{n}\sum_{i=1}^{n}{\left(y_{i}-\hat{y_{i}}\right)}^{2}}$$

(e) Overfitting Assessment

Overfitting occurs when a model performs well on training data but lacks generalization capability. Essentially, the model memorizes training data instead of learning predictive patterns, leading to poor performance on validation data. In this study, overfitting risk is evaluated using multi-dimensional metrics and visualization methods, ensuring the model’s reliability in practical LAHAM applications. A core metric is the difference in R^2^ between training and validation sets ($\Delta R^{2}$):

(7) $$\Delta R^{2}=R_{\mathrm{train}}^{2}-R_{\mathrm{val}}^{2}$$

$\Delta R^{2}>0.1$ indicates potential overfitting, reflecting excessive learning of noise in the training data.

**Figure 3:**
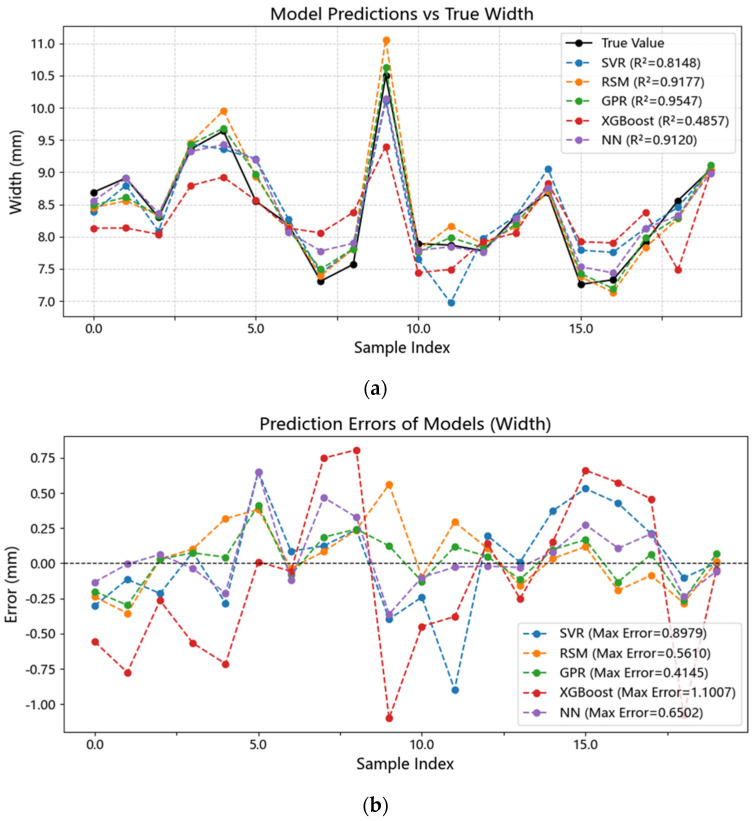
Comparison between predicted and experimental bead width using SVR, GPR, and the baseline model. GPR shows the smallest dispersion and the highest R^2^, indicating superior generalization for the nonlinear parameter–geometry mapping, while SVR exhibits moderate deviations. (**a**) Predicted versus experimental bead width. (**b**) Prediction errors of each model.

**Figure 4:**
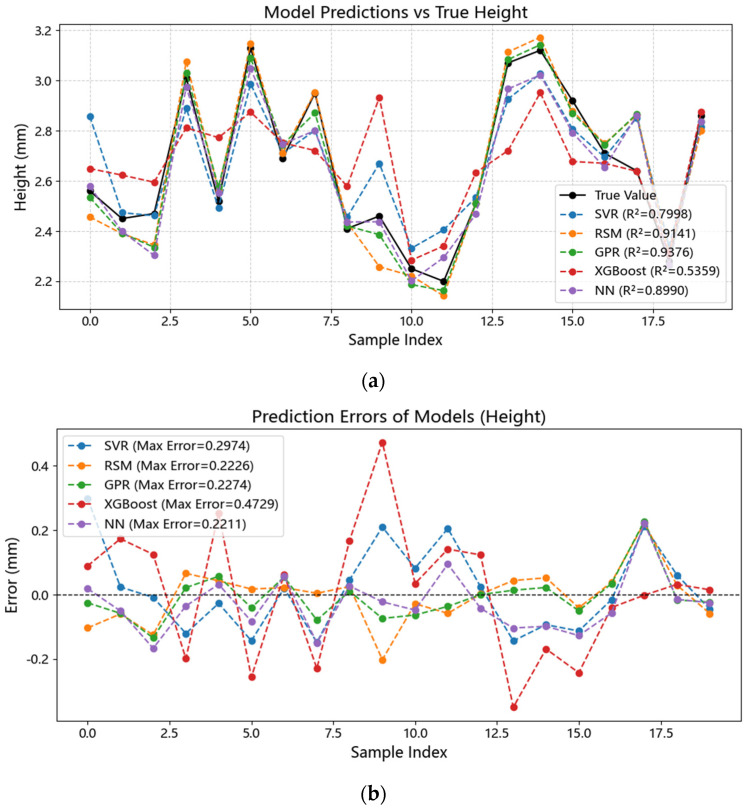
Comparison between predicted and experimental bead height using SVR, GPR, and the baseline model. GPR shows the smallest dispersion and the highest R^2^, indicating superior generalization for the nonlinear parameter–geometry mapping, while SVR exhibits moderate deviations. (**a**) Predicted versus experimental bead height. (**b**) Prediction errors of each model.

Five models—SVR, GPR, NN, Response Surface Methodology (RSM), and XGBoost—were comprehensively compared in terms of predicting bead width (W), bead height (H), and cross-sectional area (BCSA). Model performance was evaluated using R^2^, MAE, MAPE, and RMSE (Table 1), with R^2^ as the primary selection criterion. Figure 2, Figure 3 and Figure 4 illustrate the results. GPR achieved the best performance across W, H, and BCSA, particularly for W prediction, achieving R^2^ = 0.955 and MAE = 0.145 mm on the validation set, indicating strong capability in capturing nonlinear relationships. Linear models such as RSM could fit general trends but failed to accurately capture the negative correlation between laser power and H, leading to R^2^ values 1.1–3.8% lower than GPR. XGBoost exhibited severe overfitting under small-sample conditions, with validation R^2^ below 0.6, making it unsuitable for the current dataset.

**Figure 5:**
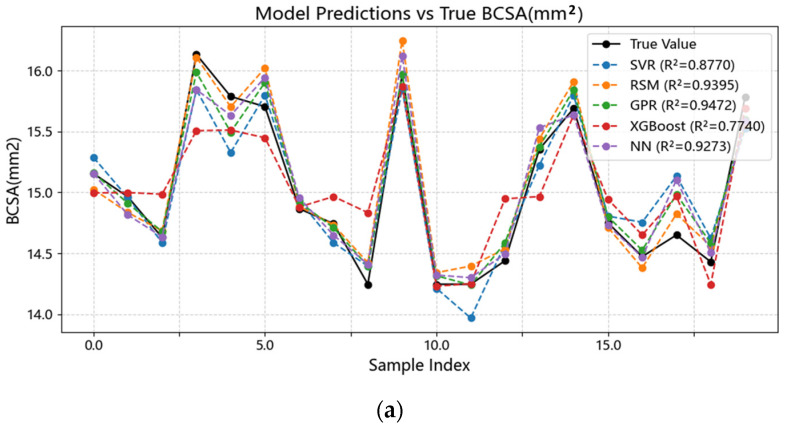

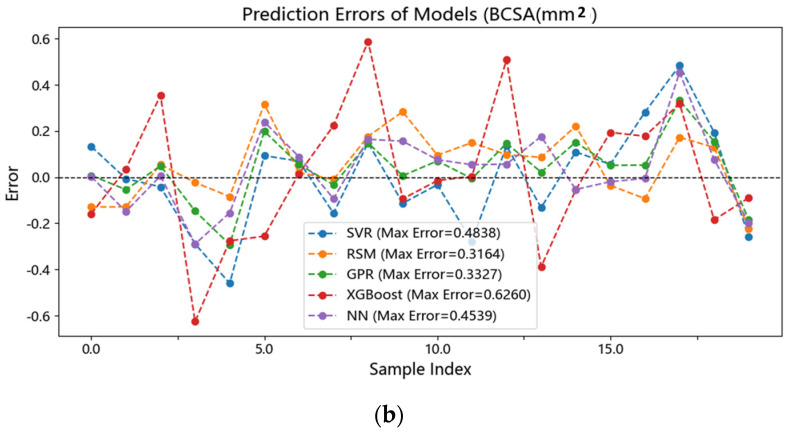
Comparison between predicted and experimental bead BCSA using SVR, GPR, and the baseline model. GPR shows the smallest dispersion and the highest R^2^, indicating superior generalization for the nonlinear parameter–geometry mapping, while SVR exhibits moderate deviations. (**a**) Predicted versus experimental bead BCSA. (**b**) Prediction errors of each model.

The GPR models demonstrated good performance on both training and validation sets in predicting width, height and BCSA (as shown in Figure 5). Therefore, GPR was selected for further hyperparameter optimization to obtain more accurate forward models for geometric parameter prediction, facilitating subsequent inverse process optimization. 

Two optimization algorithms, Bayesian Optimization (BO) and Particle Swarm Optimization (PSO), were applied. Key performance comparisons are shown in Table 2. For bead width (W), Bayesian Optimization slightly outperformed in R^2^ and MAPE, while PSO achieved slightly lower MAE; the performance difference was less than 0.3%, indicating strong robustness. For bead height (H), PSO outperformed Bayesian Optimization in all metrics, especially reducing MAPE by 0.11%, attributable to PSO’s higher efficiency in searching small-scale parameters (e.g., noise level 10^−5^).

**Figure 6:**
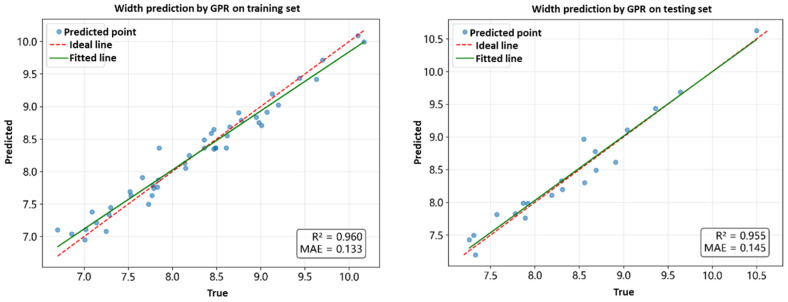

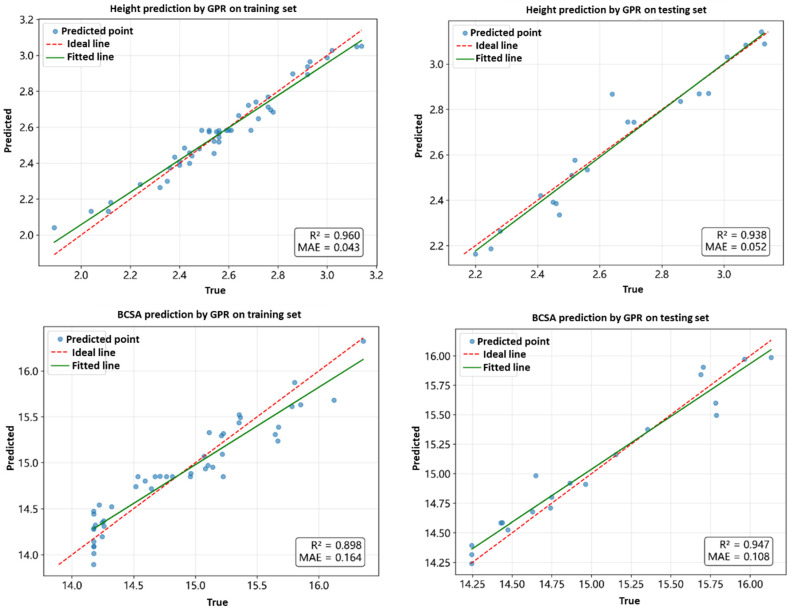
GPR performance in predicting width, height and BCSA.

Based on above, Bayesian Optimization was selected for W and PSO for H. Both models showed no overfitting, with prediction curves better matching the dataset compared to the original GPR models. Visualization of five validation points (as shown in Figure 6) confirmed significant improvement in prediction accuracy. The ΔR^2^ for both Bayesian and PSO-optimized models was below 0.01, indicating good generalization and low overfitting risk. The optimized GPR models provide reliable forward predictions of LAHAM geometric parameters. The main optimized hyperparameters for each target variable are summarized in Table 2:

**Table 2:** Main optimized hyperparameters.

Target Variable	Kernel Function Type	Length Scale	Noise Level	Alpha	Optimizer Restart Count
Width	RBF	0.1025	0.0002	4.0614 × 10^−6^	5
Height	Matern1.5	1.5561	0.0006	1.1373 × 10^−7^	11

In summary, the Bayesian-optimized GPR model was chosen for W (R^2^ = 0.9547) and the PSO-optimized GPR model for H (R^2^ = 0.9393), achieving high prediction accuracy and strong generalization capability. These models provide a robust basis for subsequent collaborative optimization of part geometry and energy efficiency in LAHAM processes.

## 4. Multi-Objective Collaborative Optimization

Achieving simultaneous optimization of geometric accuracy and energy efficiency is of critical importance in LAHAM. In this study, bead width deviation and height deviation are selected as the optimization metrics for forming quality, while the DVUE is used to characterize the deposition volume per unit energy, providing a more accurate measure of energy utilization efficiency. The optimization objective is designed to optimize the process parameters so that forming accuracy is maintained while maximizing energy utilization efficiency and reducing overall production costs. The process parameter ranges are set as follows: wire feed speed 6–8 m/min, travel speed 500–700 mm/min, arc length correction 5–15%, pulse correction 0–2 levels, and laser power 1–3 kW. The optimization is performed using the NSGA-II algorithm with a population size of 200, a maximum of 300 generations, a simulated binary crossover (SBX) operator with distribution index 20, and a polynomial mutation operator with a mutation rate of 20%, ensuring both stability and efficiency in global search.

### 4.1. Non-Dominated Sorting Genetic Algorithm (NSGA-II)

NSGA-II, proposed by Deb et al. in 2002 [34], is a significant improvement over the original NSGA and has become one of the most widely used algorithms in multi-objective optimization. Its core advantages derive from three mechanisms: fast non-dominated sorting, crowding distance calculation, and elitism preservation. These mechanisms enable the algorithm to efficiently approximate the Pareto front while maintaining population diversity, providing effective trade-offs among conflicting objectives. The fundamental principle is to use dominance relationships to rank solutions: if solution A is no worse than solution B in all objectives and better in at least one, then A dominates B. Solutions not dominated by any others constitute the non-dominated set. NSGA-II assigns rank to the population based on non-dominance, with lower ranks indicating higher overall quality. For instance, in this study, a solution with smaller deviations in both bead width and height than another solution occupies a higher Pareto rank.

The crowding distance mechanism further ensures uniform distribution within the same rank by quantitatively measuring the spacing between solutions, preventing excessive clustering of individuals in the optimal region. Boundary solutions are assigned an infinite crowding distance to guarantee their retention, maintaining the breadth of solution coverage. The elitism strategy preserves superior individuals from both parent and offspring populations across generations, preventing the loss of high-quality solutions and accelerating convergence toward the Pareto front.

**Figure 7:**
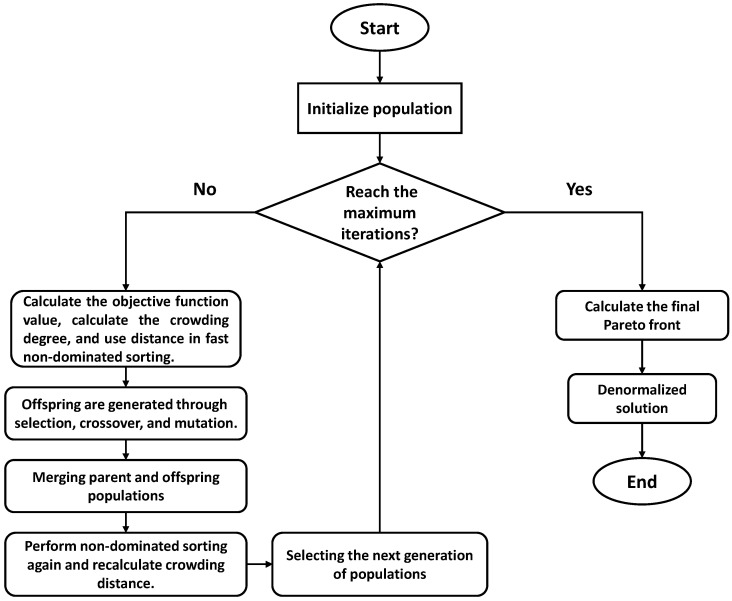
NSGA-II Flowchart.

The NSGA-II procedure for process parameter optimization in this study is illustrated in Figure 7. Process parameters (e.g., wire feed speed, travel speed) are encoded as individuals to form an initial random population. Fast non-dominated sorting determines the rank of each individual, and crowding distance evaluates distribution uniformity. Based on these criteria, a tournament selection identifies superior individuals, and SBX introduces controlled variation to enhance the ability to escape local optima. Elite merging generates the next generation population. Through multiple iterations, NSGA-II effectively balances competing objectives such as geometric accuracy and energy consumption, providing a stable and reliable optimization framework for complex process parameters.

The NSGA-II algorithm was implemented with a population size of 200, which offers a good balance between solution diversity and computational efficiency for the multi-parameter optimization problem. The maximum number of generations was set to 300 to allow sufficient convergence toward the Pareto front, considering the nonlinear coupling of process parameters. Simulated Binary Crossover (SBX) was used with a distribution index of 20, enabling controlled local refinement by generating offspring solutions close to their parents. Polynomial mutation was applied with a mutation rate of 0.2, which introduces necessary randomness for escaping local optima while preserving high-quality genes. The algorithm stopped upon reaching the maximum number of generations, which consistently produced a stable and well-distributed Pareto front.

### 4.2. Collaborative Optimization of Forming Dimensions and Energy Efficiency

In LAHAM, the collaborative optimization of forming accuracy and energy consumption is a key objective. In this study, the deviations of bead width and height are chosen as geometric quality indicators, while DVUE represents energy efficiency. In this study, DVUE can be calculated as:

$$\mathrm{DVUE}=\;\frac{BCSA\times Travel\;Speed}{P_{laser}+UI}$$

The Deposition Volume per Unit Energy (DVUE) is introduced to quantify energy utilization efficiency in the LAHAM process. The numerator, BCSA×Travel Speed, represents the effective deposition volume rate, where BCSA corresponds to the bead cross-sectional area and the travel speed determines the rate of material laying along the welding path. The denominator, $P_{\mathrm{laser}}+UI$, denotes the total energy input rate from the hybrid heat sources, including the laser power and the product of arc voltage and current. Therefore, DVUE physically describes the volume of deposited material obtained per unit of delivered energy.

The optimization goal is to minimize W and H deviations while evaluating DVUE on the Pareto front. By integrating the trained GPR models with NSGA-II, a “parameters–accuracy–energy” optimization framework is established, as follows:

(1) Objective Function Definition

Geometric accuracy objectives aim to minimize bead width deviation $f_{W}$ and height deviation $f_{H}$:

(8) $$f_{W}=\left|W_{pred}-W_{target}\right|,\;f_{H}=\left|H_{pred}-H_{target}\right|$$

where $W_{\mathrm{pred}}$ and $H_{\mathrm{pred}}$ are predicted by the GPR model, and $W_{\mathrm{target}}$ and $H_{\mathrm{target}}$ are the desired target values. The target values $W_{\mathrm{target}}$ and $H_{\mathrm{target}}$ were defined as the average bead width and height measured using a laser scanner from the baseline deposition experiments. These experimentally measured values provide realistic geometric targets for the optimization process.

The energy objective is achieved by maximizing DVUE along the Pareto front. Higher DVUE values indicate greater effective material deposition per unit energy input, thus improving energy efficiency while maintaining forming accuracy.

(2) Parameter space and constraints

Reasonable selection of process parameters is crucial. Considering equipment limitations and ensuring deposition continuity, the ranges are: wire feed speed 6–8 m/min, travel speed 500–700 mm/min, arc length correction 5–15%, pulse correction 0–2 levels, and laser power 1–3 kW. These ranges are based on prior experiments, material properties, and equipment capabilities.

(3) NSGA-II parameter settings

The population size is set to 200 to adequately explore the solution space while maintaining computational efficiency. A maximum of 300 generations allows the algorithm sufficient time to converge to the Pareto front, capturing the complex coupling of LAHAM process parameters. SBX uses a distribution index of 20 to maintain stability and gradual convergence, while polynomial mutation with a 20% mutation rate balances exploration and exploitation, avoiding premature convergence without disrupting high-quality genes.

### 4.3. Optimization Results and Discussion

**Figure 8:**
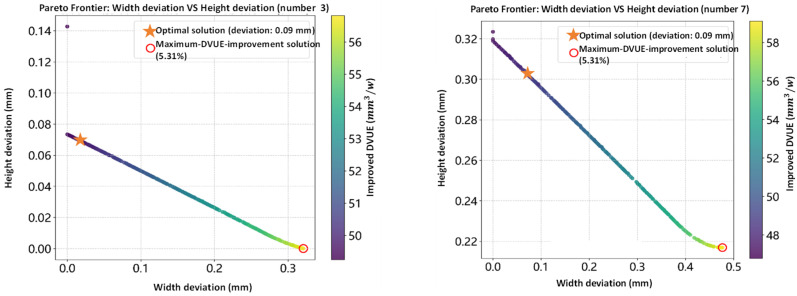

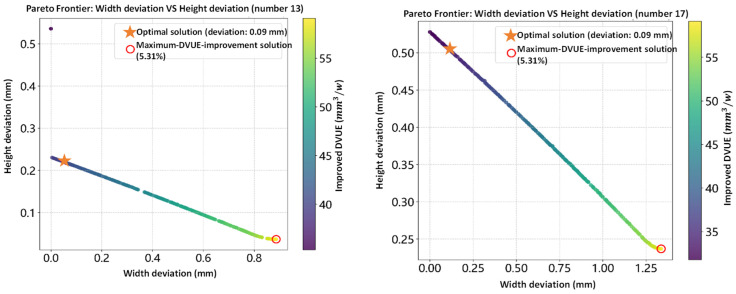
Pareto front obtained from NSGA-II optimization, illustrating the trade-off between bead width deviation, bead height deviation, and DVUE (Deposition Volume per Unit Energy). Each point represents a feasible process parameter combination. Solutions toward the lower-left region correspond to simultaneously reduced dimensional deviations and improved energy efficiency. The highlighted optimal point (selected based on knee-point criteria) shows a balanced solution achieving minimal geometric error while maintaining high DVUE.

For the testing dataset, the NSGA-II-optimized Pareto front for different testing samples (number 3, number 7, number 13 and number 17) is presented in Figure 8, with width deviation on the x-axis, height deviation on the y-axis, and DVUE indicated by color. The results demonstrate a range of trade-off solutions between geometric accuracy and energy efficiency. Each Pareto front contains solutions minimizing geometric deviation and maximizing DVUE.

Table 3 summarizes the results of the geometric optimal solutions. Taking testing sample No. 3 as an example, the target geometric dimensions are a width of 8.3 mm and a height of 2.47 mm. After optimization, the geometric-optimal solution achieves nearly ideal shape accuracy, with the width deviation reduced to approximately 0 mm and the height deviation to 0.07 mm, at the expense of a slight decrease in DVUE (−8.34%). In contrast, the DVUE-optimal solution yields a width deviation of 0.32 mm, a height deviation of 4.35 × 10^−7^ mm, and a DVUE improvement of 5.44% (Table 4). These comparisons indicate that NSGA-II is capable of significantly improving DVUE while maintaining acceptable geometric accuracy across different initial conditions, effectively balancing energy efficiency and forming precision in the optimization process.

## 5. Conclusions

This study established a machine learning-driven framework to optimize bead geometry and energy efficiency in LAHAM. Gaussian Process Regression (GPR) proved to be the most effective model, accurately predicting bead width and height with R^2^ values of 0.9547 and 0.9393 after hyperparameter optimization. By integrating the optimized GPR models with the NSGA-II algorithm, we achieved simultaneous multi-objective optimization of geometric accuracy and energy efficiency. The results demonstrate that this approach can significantly improve energy efficiency (DVUE) by over 8% in some cases while maintaining geometric deviations within acceptable limits.

This data-driven strategy provides a scalable and efficient method for guiding process design in LAHAM, balancing quality and energy use. It also establishes a foundation for future expansion into real-time control and the optimization of additional properties like microstructure and mechanical performance.

## Figures and Tables

**Table 1:** Comparison of BO-GPR and PSO-GPR for W and H prediction.

Evaluation Indicators	BO-GPR		PSO-GPR	
	W	H	W	H
R^2^	0.9547	0.9376	0.9518	0.9393
MAE	0.1445	0.0522	0.0320	0.0493
MSE	0.0301	0.0522	0.0320	0.0051
MAPE	1.7480	2.0028	1.7177	1.8888

**Table 3:** Geometric optimal solution results.

Number	Targets		Process Parameters					Prediction		Deviation		DVUE Increment /(%)
	W	H	$V_{w}$	$V_{t}$	L	f	p	W	H	W	H	
3	8.3	2.47	8	515	5	0.30	1	8.30	2.40	0.00	0.07	−8.34
7	8.19	2.69	8	522	5	0.29	1	8.19	2.37	0.00	0.32	−15.49
13	7.78	2.51	8	545	5	0.37	1	7.80	2.28	0.02	0.23	−26.12
17	7.33	2.71	8	567	5	0.03	1	7.33	2.18	0.00	0.53	−42.02

**Table 4:** DVUE optimal solution results.

Number	Deviation of Width /(mm)	Deviation of Height /(mm)	DVUE Increment /(%)
3	0.32	4.35 × 10^−7^	5.44
7	0.48	0.22	5.50
13	0.89	0.04	8.41
17	1.34	0.24	8.59

## Data Availability

The original contributions presented in this study are included in the article. Further inquiries can be directed to the corresponding author.

## Appendix A. Training Dataset

**Number**	**Wire-Feed** **Speed** **/(m/min)**	**Travel Speed** **/(mm/min)**	**Arc Length** **Correction** **/(%)**	**Pulse** **Correction** **/(%)**	**Laser Power** **/(kW)**	**W** **/(mm)**	**H** **/(mm)**	**BCSA** **/(mm^2^)**
1	7	600	5	2	2	8.19	2.78	15.10
2	8	600	10	1	1	7.66	3.14	15.21
3	8	700	10	1	2	7.14	2.54	14.18
4	7	600	10	1	2	7.85	2.6	14.54
5	7	600	15	0	2	6.7	2.77	14.18
6	7	700	5	1	2	7.28	2.56	14.18
7	7	500	5	1	2	9.13	2.76	15.78
8	6	600	5	1	2	7.52	1.89	14.18
9	8	600	5	1	2	8.44	2.92	15.35
10	7	600	5	1	3	8.62	2.45	14.71
11	7	700	10	1	3	7.09	2.11	14.18
12	7	600	10	2	3	9.63	2.12	15.07
13	6	600	10	1	3	7.3	2.32	14.18
14	6	600	15	1	2	6.86	2.76	14.19
15	8	600	10	1	3	9.7	2.35	15.67
16	7	600	10	1	2	8.36	2.52	14.67
17	7	700	10	1	1	7.53	2.55	14.26
18	7	500	10	1	3	10.1	2.56	15.85
19	7	600	5	1	1	9.01	2.42	15.14
20	6	700	10	1	2	7.25	2.54	14.18
21	7	600	15	2	2	8.75	2.72	15.65
22	6	500	10	1	2	7.79	2.56	14.52
23	7	500	10	2	2	9.44	2.86	15.80
24	7	700	10	2	2	7.83	2.38	14.26
25	8	600	15	1	2	8.78	2.52	15.11
26	7	600	10	2	1	8.14	2.92	15.22
27	6	600	10	2	2	7.84	2.48	14.32
28	8	600	10	2	2	8.65	2.68	15.36
29	7	700	10	0	2	7.02	2.44	14.18
30	7	700	15	1	2	7.73	2.4	14.25
31	6	600	10	1	1	7.78	2.36	14.25
32	7	600	15	1	3	9.2	2.04	14.59
33	7	600	10	0	3	8.47	2.24	14.22
34	7	600	5	0	2	8.95	2.44	14.96
35	7	600	10	1	2	8.49	2.61	14.96
36	7	600	15	1	1	7.77	2.93	15.08
37	7	500	10	0	2	8.47	2.71	15.23
38	8	600	10	0	2	8.98	2.64	15.67
39	7	500	10	1	1	8.36	3	15.35
40	7	600	10	1	2	8.61	2.49	14.77
41	7	600	10	0	1	8.15	2.56	14.64
42	8	500	10	1	2	10.17	3.02	16.36
43	6	600	10	0	2	7.01	2.4	14.18
44	7	600	10	1	2	8.49	2.59	14.81
45	7	500	15	1	2	9.07	3.12	16.12
46	7	600	10	1	2	8.49	2.69	15.22

## Appendix B. Testing Dataset

**Number**	**Wire-Feed** **Speed** **/(m/min)**	**Travel Speed** **/(mm/min)**	**Arc Length** **Correction** **/(%)**	**Pulse** **Correction** **/(%)**	**Laser Power** **/(kW)**	**W** **/(mm)**	**H** **/(mm)**	**BCSA** **/(mm^2^)**
1	6.5	510	6	1.5	1.2	8.69	2.56	15.15
2	6.3	540	8	1.7	2.7	8.91	2.45	14.96
3	6.6	570	7	0.2	2.2	8.3	2.47	14.63
4	7.9	530	7	0.9	1.8	9.36	3.01	16.13
5	7.2	520	6	0.3	2.9	9.64	2.52	15.79
6	7.8	510	12	0.5	1.5	8.55	3.13	15.70
7	7.3	620	12	1.2	1.6	8.19	2.69	14.87
8	6.3	580	14	1.1	1	7.31	2.95	14.74
9	7	680	14	1.3	2	7.57	2.41	14.25
10	8	540	14	1.1	2.8	10.5	2.46	15.96
11	6.3	610	6	0.5	2.3	7.89	2.25	14.25
12	6.3	660	6	0.6	1.5	7.87	2.2	14.25
13	6.6	570	10	0.2	2	7.78	2.51	14.44
14	6.8	550	14	1.6	1.2	8.31	3.07	15.35
15	7.3	520	11	1.8	1.3	8.68	3.12	15.69
16	6.7	550	13	0.3	1.2	7.26	2.92	14.75
17	7.9	690	10	0.8	1.2	7.33	2.71	14.47
18	7.3	620	7	1.9	1	7.92	2.64	14.65
19	6.9	640	9	1.6	2.7	8.56	2.28	14.43
20	7.1	520	7	1.3	1.8	9.04	2.86	15.78
12	6.3	660	6	0.6	1.5	7.87	2.2	14.25
13	6.6	570	10	0.2	2	7.78	2.51	14.44
14	6.8	550	14	1.6	1.2	8.31	3.07	15.35
15	7.3	520	11	1.8	1.3	8.68	3.12	15.69
16	6.7	550	13	0.3	1.2	7.26	2.92	14.75
17	7.9	690	10	0.8	1.2	7.33	2.71	14.47
18	7.3	620	7	1.9	1	7.92	2.64	14.65
19	6.9	640	9	1.6	2.7	8.56	2.28	14.43
20	7.1	520	7	1.3	1.8	9.04	2.86	15.78
